# COVID-19 unanticipated benefits to hand washing coverage and practices in health care facilities in central Uganda

**DOI:** 10.4314/ahs.v23i4.18

**Published:** 2023-12

**Authors:** Noe Nassolo, Abel Wilson Walekhwa, Frank Gramsen Kizza, Jimmy Osuret

**Affiliations:** 1 Department of Disease Control and Environmental Health, School of Public Health, College of Health Sciences, Makerere University. P.O. BOX 7072, Kampala, Uganda; 2 Disease Dynamics Unit (DDU), Department of Veterinary Medicine, University of Cambridge, United Kingdom, CB3 0ES

**Keywords:** Hand washing coverage, health workers, private, public, Uganda

## Abstract

**Introduction:**

Hand hygiene in health care facilities (HCFs) remains a significant public health challenge. Global baseline estimates on water, Sanitation, and Hygiene (WASH) in HCFs indicate that 26% of HCFs lack access to an improved water source on the premises. In this study, we sought to assess the proportion of handwashing coverage and the associated factors among healthcare workers in public and private healthcare facilities in Ndejje division, Makindye Ssabagabo municipality, Wakiso district.

**Methods:**

A descriptive cross-sectional study with both quantitative and qualitative methods of data collection was conducted. A total of 350 healthcare workers were interviewed using a self-administered structured open-ended paper questionnaire and Focus Group Discussions (FGD) guide. Data was analysed using STATA 14.2 and ATLAS. ti version 8 software.

**Results:**

The majority of 350 (92.6%) of healthcare workers were from private health facilities. We found out that the proportion of handwashing facilities coverage was 97.7%. The proportion of handwashing was good coupled with a positive attitude towards handwashing. Being a nurse was highly associated with washing hands in both private and public health facilities.

**Conclusion:**

High hand washing proportion was attributed to the COVID-19 guidelines and enforcement which sparked adherence to the standard operating procedures.

## Background

The Global baseline report in 2019 on Water Sanitation and Hygiene (WASH) in healthcare facilities by World Health Organisation (WHO) showed that 74% of healthcare facilities globally had basic water services, meaning water was available from an improved source on the premises (WHO 2020). Hand hygiene in health care facilities (HCF) remains a significant public health challenge (Kayiwa, Mugambe et al. 2020). Global baseline estimates on water, Sanitation, and Hygiene (WASH) in HCFs indicate that 26% of HCFs lack access to an improved water source on the premises, 14% of HCFs have a limited water supply and 12% have no water supply at all (WHO 2020). Water service indicators are worse in low-income countries (LICs) where 45% of HCFs do not have access to basic water supply (WHO 2020). About 16% of HCFs globally also lack hand hygiene facilities at points of care, in addition to lacking soap and water at toilet facilities (WHO 2017). Additionally, in LICs, HCFs lack reliable access to water, sanitation, and hygiene (WASH) infrastructure. Consequently, health workers are unable to wash their hands at critical points during service delivery. Inadequate WASH compromises the safety and quality of healthcare services and places a huge preventable risk to both health providers and clients (Bouzid, Gumming et al. 2018) for example these deficiencies in WASH increase the risk of health facility-acquired infections (HAI). Healthcare-associated infections (HAIs) affect 1.4 million patients at any time worldwide (Kayiwa, 2020), as estimated by the World Health Organization (WHO) (Cardo, Dennehy et al. 2010, Murphy, Tchetchik et al. 2020).

Health care providers' hands are the main usual mode for the transmission of HCAIs (WHO 2016). About 50% of HCAIs happens due to the hand of health care providers (Albright, White et al. 2018). During patient care, unless there is recommended hand hygiene compliance of health care suppliers uninterrupted, hands are contaminated with a microorganism (Rynga, Kumar et al. 2017). Due to poor hand hygiene practices among health care workers, many patients have suffered from HCAIs (Engdaw, Gebrehiwot et al. 2019). Improper hand hygiene by HCWs is responsible for over 40% of health facility infections (Rynga, Kumar et al. 2017) even in health facilities within the Ndejje division. These infections are also responsible for nearly 50% of the deaths that occur among patients and health care workers (Rynga, Kumar et al. 2017). In 2012, the most likely estimate of disease burden from inadequate hand hygiene amounted to 297 000 deaths (Prüss-Ustün, Bartram et al. 2014).

In Uganda, there is limited data on WASH in HCFs, however, a study carried out in the southwestern region of the country highlights that Only 38% of the HCFs had wards with handwashing facilities with only 24% of the wards having soap and water (Mulogo, Matte et al. 2018). Various health facilities in the areas are well stocked with handwashing facilities with water and soap however the actual practice of handwashing was lacking (Brauer, Zhao et al. 2020). Therefore, this study aimed to assess the proportion of handwashing facility coverage among health care workers in private and public health facilities in Ndejje division, Makindye Ssabagabo municipality Wakiso district.

## Methods

### Study area

The study was carried out in Ndejje division of Makindye Ssabagabo Municipal Council, this is in Wakiso district in Central Uganda Kyaddondo County immediately south of Kampala's Makindye division and physically lies at 0014′34.0N,320 33′36.0″E (latitude:0.242789; longitude:32.559987). The municipality includes the following neighbourhood; Mutundwe, Najjanankumbi, Zana, Bunnamwaya, Seguku, Lubowa, Ndejje, Lubugumu, Busaabala, Masajja, Kaazi, Lweza, Kigo, and Kubbiri. Makindye Ssabagabo municipality is one of the fastest-growing municipalities in Uganda and during the national census and household survey of 27 and 28 August 2014, the Uganda Bureau of Statistics (UBOS), enumerated the population of Makindye Ssabagabo Municipality at 284,067 with the highest percentage being the youths. It is the highest densely populated urban centre in Uganda with a fertility rate of 6 children. The population of the males is 132,666 males (46.8%) and that of the females is 150,606 (53.2%) (Uganda Bureau of Statistics 2017).

Ndejje division is made up of 3 wards and 19 cells whereby Seguku ward has 5 cells, Mutungo with 6 cells, and Ndejje ward with 8 cells. There are 3 public and 57 private health care facilities dispersed in all the three wards whose accessibility was by Boda boda with the guidance of the health inspector.

### Study design

A descriptive cross-sectional study design with a convergent parallel design approach was utilized. The study targeted all healthcare workers within registered and non-registered, private and public health facilities in Ndejje division, Makindye Ssabagabo municipality.

### Study population

Different health care workers including medical clinical officers, midwives, health assistants, nurses, health inspectors, medical Lab technicians, medical Lab technologists, medical radiographers, and pharmacy assistants participated in our study. A list containing all the 52 registered health facilities was obtained from the Division Health Department where they are registered and regulated to aid the sampling process. All the 3 public facilities in the Ndejje division were involved in the study and these included; Mutungo HC II, Sseguku HC II, and Ndejje HC IV together with 38 private facilities. Each private clinic had either 1or 2 staff, HC II had 4 staff each, HC 1V had 18 staff each, and hospitals had an average of 40 staff each. All the FGDs of health care workers from public health facilities had 8 and 9 participants while those for health care workers from private facilities had 5-7 participants. Healthcare workers in public health facilities together with those in big private health facilities had working schedules and shifts while those who were in small medical clinics, pharmacies, dental clinics, and drug shops worked full-time. There are two hospitals and an estimate of 60 private clinics.

### Sample size determination

The sample size was determined using the single population proportion formula (Kish 1965) for determining sample size in cross-sectional studies, $N=\frac{Z^{2}p(1-p)}{d^{2}}$ and a total of 380 health care workers. Ten (10) Focus Group Discussions with each having a total of eight participants were involved in the study with private and public health facilities each having five groups. The FGDs data was collected up to the point of data saturation.

For health facility level sampling, all the three public health facilities were purposively selected and other private health facilities from every cell constituted the sample size. With a representation of every cell, an equal number of 2 private health care facilities was conveniently selected from every cell where 38 private facilities were included in the study. Since there were few public health facilities, to draw comparative results between private and public health facilities, all public health facilities were considered together with a representation from private health facilities.

For health worker sampling, each private clinic had either 1 or 2 staff, HC II have 4 staff each, HC 1V have 18 staff each and hospitals had 40 staff each, a random stratified proportionate simple sampling procedure was applied in the selection of study participants from both public and private health facilities. Within each health facility, all health care workers were given equal chances to participate in the study through simple random sampling. A unique number with consideration of the initials was done for all health workers. This was followed by the research assistants and/or the principal investigator randomly picking a representation of the health care workers from the staff in each facility.

FGD participants were purposively selected basing on their active role in WASH at the health facility level. These included leaders of health facilities/In-charges, health care workers, private clinics owners, and supervisors. The participants were of different professional cadres, age groups, work experience and they were selected about the facilities they operated. All FGDs were conducted outdoors in the compound under tree shades where COVID-19 prevention guidelines would be properly implemented. Mutungo HC II, Sseguku HC II, and Ndejje HC IV were the venues for the public health care workers while the Makindye division Headquarters was the venue for private health care workers

### Data collection tools

Focus Group Discussion guide was used in the collection of data on the attitudes of health care workers on hand hygiene together with the associated factors. Data was collected physically by research assistants asking different questions to the respondents and the data was recorded using an audio recorder and then transcribed into notes. The research assistants were trained for 3 days by the principal investigator and were required to have a minimum of ordinary level education as a qualification. These FGDs were heterogeneous involving different staff of different professions sex, age and from different health care facilities. The homogeneity in the FGD was that an FGD for health workers from private did not by any means include an individual from public health facilities and vice versa.

**Structured questionnaire:** a semi-structured questionnaire with both open-ended and closed-ended questions was used. This was adopted from a former study by Ekanem et al 2015 and edited to suit the current study. This was mostly used when collecting the primary data from the health care workers at the health facilities on the proportion of handwashing and its associated factors. The process of data collection took approximately 20 minutes per respondent. Structured observational checklist: an observation checklist was developed by the study P.I that was used by the Research assistants to check on the practices and prevalence of handwashing. This tool had simple observational questions with Yes and No as the responses. A researcher and research assistants used this tool by observing the health facility's handwashing facility coverage, their state, and the compliance of health care workers in hand washing. Data collection using an observational checklist took an average of 5 minutes per session.

Notes taken together with audio recording was done in the assessment of factors associated with hand hygiene in private and public health facilities. A smartphone was used in the recording of the sessions. Later these audios were transcribed verbatim.

### Data collection

Before data collection started, pre-testing of all the data collection tools was done in Entebbe municipal council with in health facilities with similar characteristic as those in the study area. Data was collected using three data collection tools, for quantitative component, questionnaire and checklist were used, and for qualitative component, FGD guide (Supplementary tool I) was used.

Every group consisted of 5-7 members and each member who was a health care worker at a given health facility was eligible to participate in the study group. Each participant was given a different code such as M1, M2, M3 among others and questions were asked by the principal investigator or any other trained research assistant. FDGs each was held for an average time of one hour within the hospital premises. To allow continuity of services at health facilities, we conducted FGDs in the health facilities premises but also health workers guided us on the potential time of less volume of clients and this was around 1300hrs British Standard Time (1600hrs East African time). For privacy and confidentiality during FGDs, the health workers guided on which locations/sites where we could have minimal disruptions. We conducted FGDs across the health workers until saturation was attained in that we could no longer get new information coming through. Even individual FGDs, we allowed the discussions to continue until members themselves felt they didn't have more information to share on this topic. To ensure robustness of the FGD results, these were only conducted by the corresponding author (NN) since she had participated in the conceptualization of the study and so, she could probe further to dig more information during FGD sessions.

For quantitative data, the questionnaire was administered to the health care worker by research assistants upon signing the written consent form which provided them time to complete it in their continent time after duty, at weekends and during meal time. The convenient time for filling the questionnaire was one week when the filled questionnaire was picked from the respective health worker. In case the selected facility or health worker refused to take part in the study, the next one was considered. In public facilities, a similar trend of systematically choosing half of the staff as study participants were applied.

### Data analysis

For quantitative data, the raw data from paper questionnaires and paper checklists were entered into EPI DATA 3 software and exported to STATA 14.2(Rabe-Hesketh and Everitt 2003) for cleaning and analysis, whereby categorical variables were summarized using frequency tables and graphs while continuous variables were summarized using means and standard deviation (SD).

For qualitative data, the codes generated were analysed for consistence and either convergence or divergence. Those that converged formed a particular theme and this was deduced by the data analysis team. Those that diverged also formed a particular theme and this was also noted. Codes were developed from objective of the study and transcribed data, and then entered the ATLAS. ti version 8 software for analysis. The software developed codes which were reviewed by the research team and enabled categorization of the study findings. Using deductive thematic analysis, the categorized data was used to develop main themes which made the results of our study.

## Results

### Social demographic characteristics

A response rate of 92.1% (350/380) was obtained for our study. Out of the 350 health care workers who participated in the study, 324 (92.6%) were from private health facilities and 26 (7.4%) from the public health facilities. Female health care workers constituted the majority 205 (58.6%), more than half 185 (52.86%) were diploma holders and majority 52.57% of the respondent were in the age group of 26-35 years (Table 1).

**Table 1:** Socio-demographic characteristics of respondents

Variable	Frequency (n=350)	Percentage (%)
Sex		
Male	145	41.43
Female	205	58.57
Professional cadre		
Nurse	117	33.43
Midwife	76	21.71
Doctor	15	4.29
Laboratory technician	51	14.57
Clinical Officer	68	19.43
Others*	23	6.57
Age in years		
18-25	79	22.57
26-35	184	52.57
56-45	53	15.14
46-55	30	8.57
56 and above	4	1.14
Education level		
Certificate	123	35.14
Diploma	185	52.86
Degree	42	12.00
Marital status		
Single	168	48.00
Married	159	45.43
Separated/Divorced/Widowed	23	6.57
Religion		
Christian	244	69.71
Muslim	71	20.29
Pentecost	31	8.86
Others**	4	1.14
**Total**	**350**	**100**

* Health inspectors, health assistants, dentists, counsellors, consultants

** Traditionalists, Born again

### Proportion of hand washing

All 350 (100.0%) health care workers in the assessed health facilities reported to be having handwashing facilities with 346 (98.9%) having their handwashing facility in good condition with soap, water and evidence of use. Over four in five of the respondents reported having Infection Prevention and Control guidelines in their facilities. (Table 2).

**Table 2:** Condition of a handwashing facility

Variable	Frequency (N=350)		Percentage (%)
Present	Yes	342	97.7
	No	8	2.3
Close to the latrine	Yes	237	67.7
	No	113	32.3
In working area	Yes	230	91.4
	No	120	34.3
Near the waste bins	Yes	198	56.6
	No	152	43.4
Inward	Yes	245	70.0
	No	105	30.0
Clean	Yes	330	94.3
	No	20	5.7
Good mechanical condition	Yes	275	78.6
	No	75	21.4
Has sock pit/container	Yes	297	84.9
	No	53	15.1
Raised above the ground	Yes	334	95.4
	No	16	4.6
Receptacle for soap	Yes	293	83.7
	No	57	16.3
Has water	Yes	335	95.7
	No	15	4.3
Has a cover	Yes	327	93.4
	No	23	6.6
Foot-operated/pedal/elble tap	Yes	188	53.7
	No	162	46.3

### Motivating factors for hand washing among healthcare workers in public and private health facilities

The major source of water for handwashing available at the health facility was tap 320 (91.4%) followed by rain 12 (3.4%), spring water 5 (1.4), and others 12 (3.7%). Among the 332 (94.9%) health care workers who had washed hands on the day of the interview, the reasons for washing hands were; to remove germs 317 (95.5%), good etiquette 4 (1.2%), to look smart/clean 4 (1.2%), and others 7 (2.1%). Health care workers washed hands as a result of water availability on the health facility premises, sufficient time for washing hands, distance to the handwashing facility, and operability of the facility.

From qualitative findings, the following factors were recorded;

From qualitative findings, it was reported that the proportion of handwashing coverage together with the compliance of health care workers to hand washing was exceedingly high in Ndejje division, Makindye Ssabagabo municipality since it's a prevention measure for the current pandemic of COVID-19

*“Almost every health care facility has a handwashing facility since it is a measure of preventing COVID-19”* (FGD, private facility N)

On the other hand, hand washing with water only together with handwashing with soap was reported as being replaced using alcohol-based hand sanitizers. This practice has also escalated within the COVID-19 pandemic and was thought to decline shortly since human behaviors change periodically most especially when the introduced behavior has not been innate.

*“Some of us no longer waste time washing hands with soap since we have our sanitizers. However, this practice of hand hygiene might not last long since we are not used to it”* (FGD, private facility M)

### Availability of hand washing supplies in various health facilities

Health care workers reported the need for handwashing materials within the health facility premises to improve their handwashing practices. Such materials included; enough hand washing cans, liquid soap, disinfectants in water, improved hand washing equipment, and a continuous supply of water. These handwashing materials were reported as factors responsible for handwashing among the health workers in Ndejje division.

*“We need more and improved handwashing facilities in all stations of the health facility so that we have separate ones for us as health care workers without sharing with the patients”* Public health facility staff, facility K

*“Rainwater harvesting techniques should be considered to solve the problem of water shortages …”* Public health facility staff, facility M

Other water additives were reported as motivators for handwashing including chlorine and/or Dettol.

*“There is a need for liquid soap or Dettol within the water used for handwashing or provision of automatic hand sanitisers”* Public health facility staff, facility O

*“Provide water with chlorine and soap”* Private health worker, facility X

### Information about handwashing

More guidelines about handwashing should be in place since it was reported as a factor that influences hand washing among health care workers. These guidelines and advises on proper handwashing were proposed to flow continuously from the supervisors to the subordinates.

*“Through maintaining the information flow of guidelines on hand washing and also advising every healthcare worker in the facility to wash hands continually by their supervisors”* will improve hand washing coverage and practices in health care facilities in central Uganda Private health worker, Facility Y

### Attitudes towards hand washing

Different perceptions were obtained on; the preference of hand sanitizers for hand washing with water, the need for information provision, and the preference for Personal Protective Equipment (gloves).

### Preference for hand sanitizers for disinfecting the hands

Health care workers reported that handwashing was at times being substituted with the use of a hand sanitizer due to its time-consuming. In addition, it was reported that handwashing with soap makes hands safer than a hand sanitizer since a sanitizer leave behind dirt and the debris.

*“I would also like to always wash my hands before and after every procedure but unfortunately due to limited time and conflicting priorities, I find myself using a hand sanitizer yet it does not make my hands 100% safe”* Private health worker, Clinic X

It was thought that private health facilities have facilities that enable them to record high hand washing practices and prevalence than the government health facilities. This was because government health facilities have their hand-washing equipment from donors and the government budget is allocated to that facility unlike the private which receive money from different sources.

*“I think we are good at hand washing than the government health facilities since, for them, their equipment relies on the national budget and donations”* Private health worker, Clinic Z

### Need for training

The need for training together with Information Education Communication (IEC) materials on handwashing might be perceived as a motivator for hand washing among health care workers.

*“I think hand washing is highly practiced in places where posters are available”* Public health facility staff, facility O

*“………. when people are educated/trained, they tend to respond accordingly though it is not an assurance”* Private health worker, Clinic N

In addition, other health care workers reported that the provision of handwashing Information Education and Communication (IEC) materials around the health facility or the handwashing facility could have contributed to the improvement of the current level of handwashing

*“I think they should put posters showing the technique of handwashing on the handwashing equipment to boost the level of handwashing among our health care workers.”* Private health worker, Clinic M

*“More posters are needed showing the 5 steeps of handwashing”* Public health facility staff, facility Y

### Preference of personal protective equipment

The provision of adequate examination gloves to ensure safety and appropriate hand hygiene was both considered as a substitute and a supplement to handwashing with soap.

*“Surgical gloves should be available to all health care workers at all times to boost hand hygiene in addition to hand washing”* Public health facility staff, facility Y.

## Discussion

Females were the majority and most of the study participants originated from private health facilities. This sociodemographic characteristic represents the current population dynamics for Uganda as a country where the females are more than males. This is in line with another study across the world by Joshi et al. where females constituted the majority totalling to greater than 50% (Joshi et al. 2013). Having more nurses in this study could be attributed to the fact that many health facilities recruit the highest number of nurses than other cadres. Our results are in agreement with this study by Abd Elaziz et al. that also showed more nurses in various health facilities than the males (Abd Elaziz and Bakr 2009, Jemal et al 2018). We also found out that most of our respondents had diploma as a highest qualification. This could be attributed to the fact many cadres who get recruited at the levels of health facilities are required to have such a level of training. This was also reported by previous study in Uganda by Jemal where a diploma was reported as a highest level of education (Jemal 2018).

The proportion of handwashing was 97.7% evidenced by the presence of handwashing facilities, 98.86 reported being having handwashing facilities in good condition. Health care workers washed hands because of water availability on the health facility premises, sufficient time for washing hands, distance to the handwashing facility, operability of the facility. in another study. This rise in the handwashing facilities coverage could be a positive effect of COVID-19 which is coupled by increased investment in preventive medicine but also increased enforcement of the COVID-19 guidelines under the presidential guidelines. Other studies in the past including that of Olum et al attributed this to efforts vested as a result of COVID-19 (Olum, Atulinda et al. 2020). According to the CDC, the single most important thing that can be done to keep from getting sick and spreading illness to others is to wash hands with soap. All stations within the health facility should make it as simple as possible for everyone to clean their hands (CDC 2018). This study recorded a higher percentage of handwashing facilities in good condition which could be attributed to the study being conducted during the second wave of COVID-19 where handwashing was highly promoted by different stakeholders as a prevention and control measure., there was an increased awareness and belief that handwashing with soap is critical to minimizing disease spread with specificity to COVID-19. Moreover, experience and literature that new behavioral patterns that emerge in response to health outbreaks or particular events do not last longer (Carver, Scheier et al. 2010). There is a need for continued maintenance of the proportion of hand-washing and the condition of the handwashing facilities.

A study highlights that over 87.5% of health workers in Nepal used the hand hygiene products available to them. In the same study, the frequency of handwashing after exposure to hospitals instruments, blood or other body fluids among the respondents was remarkably high (more than 90%) among all professionals. Similarly, hand washing practice after blowing the nose, sneezing or coughing into the hands was higher in nursing students and nurses (more than 90%) (Joshi, Joshi et al. 2013). In Cairo, it was found that the overall hand hygiene compliance among health care workers was 34% (Abd Elaziz and Bakr 2009).

The current study found that the absence of water was one of the most hindrances of hand washing among health care workers and most of them suggested alternative sources of water like rainwater harvesting. Other hindrances include the absence of soap at the station, long distance to the handwashing station, poor mechanical condition of the handwashing facility, limited time' having a hand sanitizer, and the operability of the hand-washing facility. This finding is similar to what (Jemal 2018) reported where 25.8% reported work overload, and 31.9% gave a shortage of time as a reason for not washing their hand. In addition, 28.6% complained of a shortage of water; 8.8% complained of a shortage of soap, and 5.5% complained of a shortage of antiseptic agents and scarce handwashing supplies. Similarly, another study in Ghana reported that handwashing practice is mainly affected by the availability and accessibility of handwashing facilities such as soap, towels and clean running water (Papoe M 2011). The availability of soap was not sustainable, the continuity of water supply to the handwashing facility not being satisfactory and thus significantly affected hand washing. This is similar to other studies which also recorded P-values less than 0.05 under the chi-square regression analysis (Dobe, Mandal et al. 2013, Hutton and Chase 2016). In addition, qualitative results from the current study pointed out the need for continuous supply and provision of water with soap and other disinfectants like chlorine. There is therefore a need to ensure a continuous supply of water and soap. A similar study reported that out of the total 336 participants, 159 (47.3%) reported that “appropriate placement and easy accessibility of soap dispensers and handwashing stations” could be the most important factor influencing the health care workers' compliance with hand hygiene followed by the importance of formal training on hand-washing and hygiene 149 (44.3%) and provision of liquid hand wash instead of soap bars 120 (35.7%) (Joshi, Joshi et al. 2013). Therefore, handwashing with soap should be combined with the use of a hand sanitizer to combat the likely effects of poor hand hygiene.

As hand washing was reported to be a means of preventing the transmission of germs, some health care workers reported being having no time for handwashing amidst their work schedule. In a similar study in the study agreed that hand washing could be an effective measure preventing healthcare-associated infection (Hillier 2020). Using hand hygiene as a sole measure for infection prevention and control is unlikely to be successful when other factors such as environmental hygiene, crowding, staff levels and education are present (Abd Elaziz and Bakr 2009). It was perceived that the utilization of hand gloves substitutes for handwashing. This is contrary to what the standard should be.

Many diseases and conditions can be spread by not washing hands with soap and clean running water (CDC 2016). This was this study where the majority of the respondents agreed that hand washing was important and prevents cross-contamination. Therefore, to maintain safety, dual hand hygiene should be maintained whereby health workers should wash hands in addition to the use of hand gloves. The utilization of posters to guide health care workers on timely handwashing was reported in the current study. Using posters depicting hand hygiene instructions, and senior health workers playing role models for junior colleagues were also reported in other studies (McInnes, Phillips et al. 2014). Hand washing can be promoted though hygiene education, germ-health awareness, the use of posters, leaflets, comic books, songs, and drama (Regina, 2015). In Bangladesh, posters, guide handbooks, folk songs and street plays related to health and good hygiene are among the factors used to promote and increase knowledge and practice of hygiene-related behavior such as hand washing (Akter T 2014).

## Limitations

The study used a self- reported approach during quantitative data collection, and this could have caused reporting bias at data collection in that health workers could tell what you want to hear but also what you expect them to be doing since they are expected to be role models in the community. This could portray a picture not representative of the actual situation more so on the practice of Handwashing in health facilities.

Although we think that having a high representation from private than public and therefore the findings could be skewed to the private sector but this is interesting for us since we have minimal studies that have explored this subject matter in private health facilities and therefore this shades some light on the needs for hand washing in private setting which licensing authorities like Allied Health Professionals Council and National Drug Authority for Uganda's case could explore in bid to improve infection control in such facilities.

We focused on the professional staff who are recognized by professional Councils of Uganda, and we missed important insights from low class cadres like porters, cleaners, and askaris yet they play significant role in handwashing in health facilities. Further research could explore these specific professionals since we hypothesize, they have peculiar needs for WASH that need to be paid attention to.

## Conclusion

From the general perspective, hand hygiene is the simplest method that is effective in terms of cost with its importance in infection prevention and control. Though this is the case, it was found to be high in most healthcare settings. The proportion of hand washing which was evidenced by the coverage of handwashing facilities was very high and appealing among all the health care facilities. The attitudes towards hand hygiene among health workers were generally good. Hand washing was affected by marital status, religion, professional cadre, operability of the handwashing facility, distance to the handwashing facility and the availability of sufficient water. Therefore, the sensitization on handwashing during the COVID-19 pandemic should be maintained even when the pandemic is gone to sustain the gains in handwashing coverage.

## Recommendations

There is a need to enforce hand hygiene among health care workers since the presence of handwashing facilities is not a direct reflection of hand washing. There is a need to always ensure a continuous supply of water and soap at health facilities

## Figures and Tables

**Figure 1:**
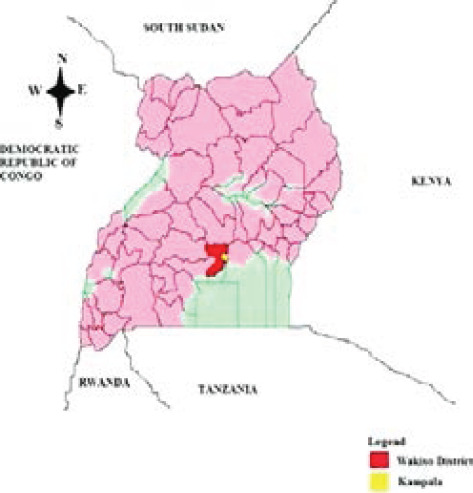
Map of Uganda showing Wakiso District

## Data Availability

The data collected for this study is readily available and can be accessed by sending a request to Ms. Noreen Nasolo, noenassolo@gmail.com

## Abbreviations

AMR: Antimicrobial Resistance
CDC: United States Centres for Disease Control and Prevention
COVID-19: Corona Virus Disease 2019
FGDs: Focus Group Discussions
HCWs: Health Care Workers
IPC: Infection Prevention and Control
MaKSPH: Makerere University School of Public Health
MOH: Ministry of Health
PPE: Personal Protective Equipment
WASH: Water, Sanitation, and Hygiene
WHO: World Health Organization
